# A Meta-Ethnography of Women’s Intimate and Sexual Experiences Across the Menopause Continuum

**DOI:** 10.2147/IJWH.S517807

**Published:** 2025-08-08

**Authors:** Samar Alotaibi, Sharron Hinchliff, Parveen Ali

**Affiliations:** 1 School of Allied Health Professions, Nursing and Midwifery, University of Sheffield, Sheffield, UK; 2 Maternal and Child Health Nursing Department, College of Nursing, Shaqra University, Al Dawadmi, Saudi Arabia; 3 Doncaster and Bassetlaw Teaching Hospitals, Doncaster, UK

**Keywords:** menopause, midlife, sexuality, intimacy, meta-ethnography, qualitative synthesis

## Abstract

Women’s intimate and sexual experiences during midlife are shaped by complex biopsychosocial factors, warranting focused attention in research and clinical practice. A hormonal shift in the menopause transition period may cause symptoms such as vaginal dryness, thinned mucosa, and painful intercourse. Other symptoms include decreased libido, and difficulty achieving orgasm during sexual activity. With more women reaching midlife, there is more need to understand how physiology and psychology impact their intimate relationships and overall health. The purpose of this review was to explore published qualitative studies on women’s sexual experiences including function, satisfaction, and challenges during midlife and the menopause continuum. A meta-ethnographic approach was conducted to synthesize all relevant qualitative studies. We searched on the Web of Science, Scopus, and CINAHL for studies published between 2010 and September 2024. Of 1,361 studies identified, 53 studies encompassing diverse cultural contexts met the inclusion criteria and were synthesized. Three main themes were identified: physical symptoms and cultural influences on sexuality during menopause and midlife; emotional, psychological, and relational responses to sexual and bodily changes, including shifts in self-perception, body image, emotional well-being, and relationship dynamics; and adapting to sexual changes during the midlife and menopause transition. The review highlights that biological changes alone are insufficient to fully understand midlife and menopausal women’s sexual experiences, emphasizing the need for a biopsychosocial approach to provide holistic care. Greater attention should be given to midlife women’s sexual health and well-being by raising awareness and encouraging open discussions about menopause and sexual changes. Healthcare professionals play a critical role in recognizing the psychological impacts of these changes and facilitating conversations to help women feel comfortable discussing sensitive topics. The review highlights the need for future research to explore women’s experiences of menopause and sexual health and well-being across diverse cultural contexts, with particular attention to non-Western countries where such perspectives remain underrepresented.

## Introduction

Menopause is considered a natural phase in a woman’s midlife that signals the end of reproductive life.^1^ It is a natural biological process that may affect their physical, mental, and sexual well-being.^2^ This involves a decline in estrogen and progesterone levels as women approach middle age and eventually cease menstruation. Menopause is diagnosed retroactively since the onset is defined as a 12-month discontinuation of menstruation.^3^,^4^

Menopause transition can last from a few months to several years, starting typically in the mid-40s to mid-50s, with the average age being 51.3 years.^5^ It is expected that over a billion women globally will be either perimenopausal or postmenopausal by 2030, and nearly 50 million women are expected to reach menopause each year.^6^,^7^ Additionally, women over the age of 50 will constitute approximately one-fifth of the global population by 2050,^8^ requiring improved healthcare resources. Menopause is not simply a physiological phenomenon; it is also a sociocultural phenomenon. Cultural and social factors play a significant role in how women perceive changes in their behavior over time.

Sexual activity is an important aspect of women’s quality of life during the menopause transition.^9^ The sexual life of middle-aged women may change due to hormonal changes,^10^ psychosocial factors^11^ and cultural influences.^12^ A common sexual problem reported during the menopause transition is vaginal dryness,^13^ resulting in dyspareunia making sexual activity more challenging for women. Additionally, low sexual desire is a common sexual concern reported by women who are undergoing the menopause transition.^14^ Sexual dysfunction leads to decreased life satisfaction and prevents satisfying sexual activities.

**Figure 1:**
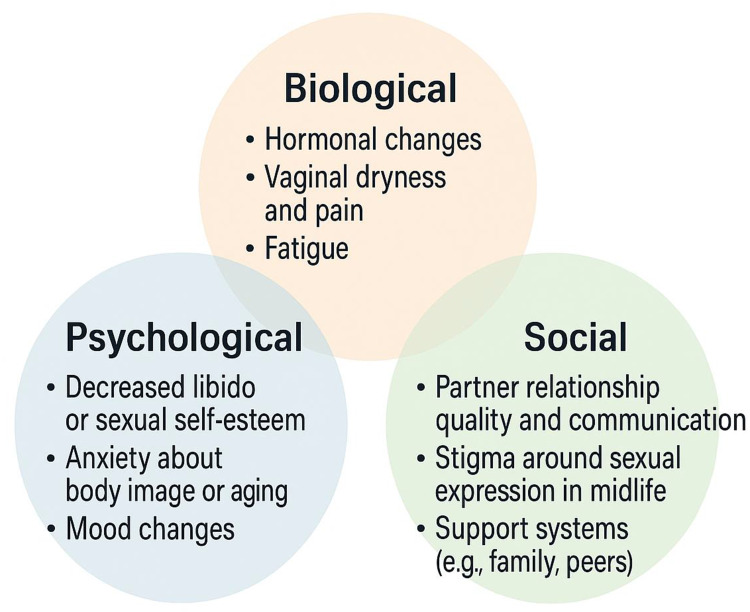
Biopsychosocial Factors Shaping Sexuality During the Menopausal Transition.

Midlife and menopause bring significant changes to women’s sexual health and overall well-being. These changes are influenced not only by biological factors but also by cultural, societal, and relational dynamics.^15^,^16^ The biopsychosocial perspective offers a comprehensive framework for understanding women’s intimate and sexual experiences during midlife and menopause by integrating biological, psychological, and social dimensions.^16–18^ Biologically, menopause is marked by hormonal changes that affect sexual functioning. Psychologically, these changes can influence self-image, emotional well-being, and desire. Socially, cultural beliefs, partner dynamics, and societal expectations shape women’s perceptions and experiences of sexuality in midlife and beyond. Figure 1 presents an example of the interrelated aspects of the biopsychosocial model as applied to women’s experiences of menopause and sexual changes. This approach is particularly important because menopause, while often framed as a biological event, occurs within the broader context of women’s lives, where factors such as caregiving responsibilities, professional demands, and societal norms play significant roles, all of which can pose emotional and relational difficulties.^16^,^19^,^20^ Biological changes, including hormonal fluctuations and ageing, are well-documented influences on sexual function and desire.^21^ However, psychological factors such as stress, fatigue, and self-perception due to bodily physiological ageing also intersect with social and relational dynamics to shape women’s experiences.^15^,^22^ Shifts in physical and mental health for women or their partners during midlife can influence both sexual well-being and overall quality of life.^23^,^24^ Additionally, social and lifestyle transitions during midlife, such as increased job demands, employment uncertainties, and evolving family responsibilities, including managing adolescent or adult children at home and caring for ageing parents, can significantly affect women’s sexual activity during this stage of life.^15^,^19^ These demands, compounded by societal expectations that idealize youth and stigmatize aging, can affect how women perceive their bodies and their sexual self-perceptions.^25^

Importantly, not all changes in sexual interest or satisfaction during this stage are directly attributable to menopause. Fatigue, stress, and relational dynamics, including the quality of partner relationships, often play critical roles.^16^,^26^ By situating sexual experiences within this complex social and cultural context, this review emphasizes the importance of a holistic approach to understanding women’s intimate lives during midlife and menopause. Exploring these aspects provides a more comprehensive understanding of women’s experiences and ensures that healthcare practices are culturally sensitive and inclusive. Therefore, this review aims to answer the question: What are the sexual experiences of women during midlife, the natural menopause transition, and beyond? The question was developed to address a gap in understanding the biopsychosocial dimensions of women’s experiences in diverse cultural contexts. Focusing on qualitative studies allows for a deeper exploration of women’s lived experiences, offering rich, nuanced insights into the complex biopsychosocial factors influencing sexuality during menopause and midlife that quantitative methods often overlook.^27^ This approach is particularly suited to capturing the personal, relational, and cultural nuances that shape women’s experiences, which are difficult to quantify through other methods. By understanding these experiences, healthcare providers can offer more holistic care with an acknowledgement of cultural differences, helping women feel supported in their personal and relationship well-being. Through this culturally sensitive approach, midlife women can gain a better quality of life and contribute to a caring healthcare environment.

## Materials and Methods

### Methods

The review was conducted following the Preferred Reporting Items for Systematic Reviews and Meta-Analyses guidelines for literature searches PRISMA guidelines,^28^ and adhered to the meta-ethnography reporting guidelines.^29^ Meta-ethnography, as developed by Noblit and Hare,^30^ is an interpretative synthesis method designed to generate new conceptual insights by analyzing and translating findings across qualitative studies. Given the study’s aim to explore women’s intimate and sexual experiences during midlife and menopause, meta-ethnography was deemed an appropriate methodology to uncover deeper meanings within the data.

Three major databases including Web of Science, Scopus, and CINHAL that cover the international health science literature were searched. Grey literature was excluded to focus on peer-reviewed studies, ensuring methodological rigor and rich qualitative data for synthesis. The question was formed broadly to check for all global qualitative studies that address sexuality in the menopause transition. The review question was developed using a PEO format which is useful for qualitative research, where population, exposure and outcomes identified. The search terms for the population labelled as “Menopause OR Climacteric OR postmenopause OR perimenopause OR midlife”. Exposure is identified as “sex OR sexuality OR sexual”. Outcomes are their experiences that are shared through “Qualitative OR interview OR focus AND group”. All these terms were combined by Boolean operator “AND”. Research is limited to studies from 2010 until September 2024. By starting the search in 2010, the review captures more recent studies, ensuring that findings reflect contemporary perspectives and social contexts relevant to women’s midlife and menopause experiences.

### Inclusion and Exclusion Criteria

The inclusion criteria for this review include any relevant qualitative data or qualitative components from mixed methods studies that explored the sexuality of menopausal or midlife women in general populations. Studies that address natural menopause and were published in peer-reviewed journals in English were included. We excluded studies that are not published in the English language, studies that focus on specific subgroups (eg, women with distinct health conditions, premature menopause, surgical menopause, specific social circumstances, or legal system involvement) to maintain a focused scope on the experiences of women undergoing natural menopause in the general population. These subgroups often face unique and complex challenges that warrant separate, focused exploration beyond the scope of this review. Studies that aimed to understand menopausal issues solely from the perspectives of men or healthcare professionals were excluded.

### Data Management and Extraction

**Figure 2:**
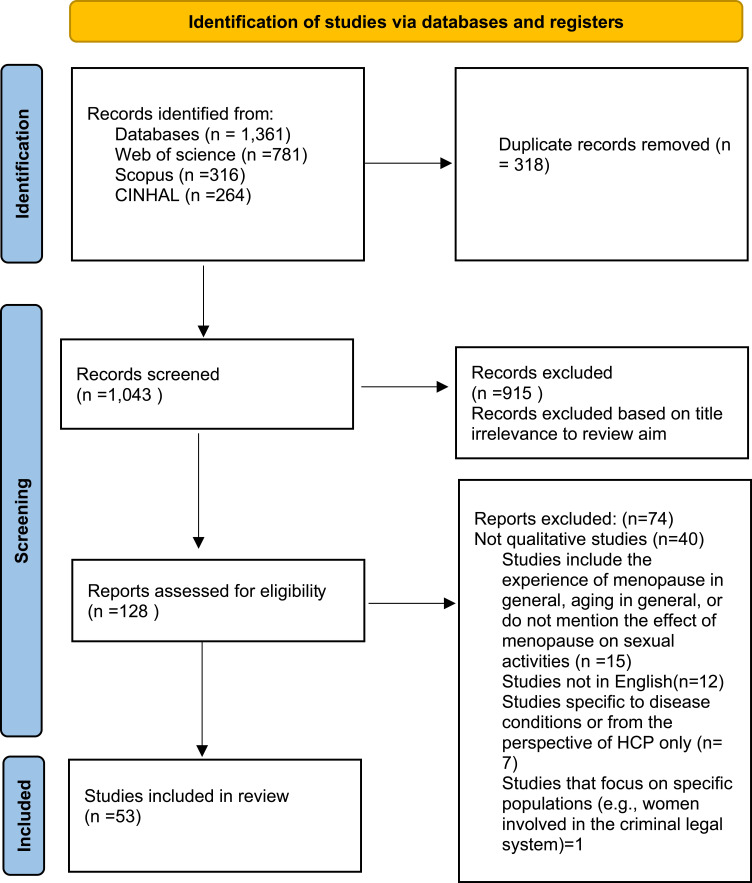
PRISMA flow diagram literature search.

In total, 1,361 studies were found and transferred to Endnote. After removing the duplicates, 1,043 were assessed for their inclusion possibility. Titles and abstracts were reviewed independently for relevance. For potentially relevant studies, full texts were retrieved and assessed for eligibility. The review excluded studies on surgical menopause or specific health conditions, focusing instead on natural menopause experiences. Studies that used quantitative methods were also excluded to maintain a qualitative focus on sexuality and relationships. In excluding specific health conditions, we were able to examine natural menopausal experiences without confounding factors. Studies that did not meet the inclusion criteria were excluded. Screening of the articles, as well as the quality assessment of the included studies, was undertaken collaboratively by all authors. Decisions regarding inclusion and appraisal were discussed and agreed upon to ensure consistency and rigour throughout the review process. A total of 53 studies that met the inclusion criteria were included. Figure 2 shows the PRISMA flow diagram of the selection process.^28^

### Quality Assessment

The CASP^31^ (Critical Appraisal Skills Program) checklist for qualitative studies was used for the risk of bias assessment. Using the CASP tool, studies were rated as high-quality if they satisfactorily addressed all or nearly all CASP criteria, moderate quality if they demonstrated limitations in two criteria, including insufficient discussion of ethical issues, lack of detail in data collection methods, inadequate consideration of researcher-participant relationships, or insufficient description of data analysis processes. Studies were rated as poor quality if they failed to meet several key criteria, such as providing insufficient detail on data collection, lack of consideration of researcher-participant relationships, and inadequate description of data analysis processes, as observed in one study in this review. There were 41 high-quality studies, 11 moderate-quality studies, and 1 poor-quality study. These results are shown in Table 1.

**Table 1:** Summary of Study Characteristics, Main Findings, and Quality Assessment Results

Author (Year of Publication), Study Location	Sample and Age Range	Study Design and Methodology	Aim of Study	CASP Quality	Main Findings
Agunbiade and Gilbert (2020), Nigeria^26^	Total: 107 older Male and female (60+)	Exploratory qualitative study (part from mixed method study). 12 vignette-based focus groups and 18 face-to face semi-structure interviews	How cultural beliefs around the woman’s body, menopause, and sexual obligations in marital relationships influence sexual behaviors and sexual rights in old age within the Yoruba context in Southwest Nigeria.	High	Many view becoming a grandmother as a marker for sexual retirement, while parental responsibilities limit sexual freedom. Symptoms like vaginal dryness are seen as reasons to stop sexual activity, with continued engagement potentially leading to social stigma.
Amini and McCormack (2021), Iran^32^	30 older women aged +45	Qualitative Face to face interviews	To understand how sexuality operates as a vector of power in Iranian culture, intersecting with gender and age, to limit women’s experiences and lives.	High	Sex is seen as a marital duty, with some women faking satisfaction during menopause. For others, menopause prompts challenges to patriarchal norms, expressing dissatisfaction, or rejecting sex. Cultural pressures encourage cosmetic interventions to satisfy husband.
Amini and McCormack (2019), Iran^33^	30 older women who aged +45	Qualitative Face to face interviews.	To examines menopause as an extended period during which women evaluate their lives in the context of the patriarchal culture of Iran.	High	Some women view menopause as a serious illness and lack knowledge about it. There were concerns about post-menopausal beauty led some to cosmetic interventions. Women vary in negotiating or hiding their sexual preferences during menopause.
Bahri et al (2017), Iran^34^	21 women aged (42–55 years).	Qualitative Semi-structured in-depth interviews.	To explore the ways of managing sexual dysfunctions during the menopausal transition among Iranian women.	High	Most women experienced a decline in libido during menopause, influenced by others’ experiences. Concerns about husbands’ unmet needs lead to obedience for religious and cultural reasons, with fears of marital conflict. Some seek support from religious teachings and peers or cope through exercise and open dialogue, though cultural norms often limit discussions.
Javadivala et al (2018), Iran^9^	22 married women ages 44–59 years.	Phenomenological approach. Face to face interviews	Explore how women assign meaning to and process sexual motivation during the menopausal transition.	High	Women report reduced sexual desire due to menopause, partner disinterest, lack of mutual understanding, and parental duties. Some feel religious and marital obligations to meet their husbands’ desires. An active, happy life and maintaining an attractive appearance are seen as motivators for sexual engagement.
Moghasemi et al (2018), Iran^35^	17 women aged 40 −65 years.	A qualitative descriptive design semi-structured in-depth	Investigate Iranian women’s attitudes and experiences about sexual life changes in midlife.	High	Most participants felt societal pressure toward asexuality, impacting their desire. Some found midlife sex more enjoyable due to experience and valued a strong relationship. Menopause signalled lost fertility for some, while others saw it as freedom from pregnancy. Adaptations included adopting youthful roles in sex or enhancing appearance with cosmetic procedures.
Azar et al (2016), Lebanon^36^	23 women aged (40–55 years)	Qualitative design, semi-structured individual interviews and focus group	To explores middle-aged Lebanese women’s understanding of sexuality and how this is shaped by the personal, sociocultural and religious context.	High	Women’s submission is shaped by sociocultural factors like economic dependence, religious beliefs, stigma, and concerns for children, with some sacrificing to satisfy husbands. Most Muslim women see sex as a religious duty. Menopause is often viewed as a hopeless age tied to reproductive limitations, though some find sexual freedom. Suggestions include delaying menopause with hormones or using plastic surgery to look younger.
Azar et al (2021). Lebanon^37^	52 women aged 40–55 years.	Qualitative design semi-structured individual interviews and focus groups	To understand the way middle-aged Lebanese women address their sexual difficulties, considering the facilitators and barriers to help seeking and the different sources of help.	High	Postmenopausal sex is low priority for some, with decreased desire linked to husband’s health. Women attribute sexual difficulties to psychological factors, seeking help primarily from husbands, followed by trusted gynecologists.
Varisoglu and Oskay (2018), Turkey^38^	15 married women 60 years or older	Qualitative study. semi-structured questionnaire applied using face to face interview method.	To identify the changes in the sex lives of women older than 60 years and to determine the effects of the advanced age on sexuality.	Low	Women in harmonious marriages report positive sexual experiences, while marital discord, lack of love, and dyspareunia negatively affect sexuality. Some women are content living as companions without sex.
Yang et al (2016), Taiwan^39^	18 married women aged 45–60.	Qualitative study In-depth interviews	To explore Taiwanese women’s perceptions of the impact of menopause on their sex lives, to assess whether the women adhered to traditional eastern.	High	Women noted decreased sexual interest due to menopause and partner health issues, though some experienced increased desire or no change post-menopause. Menstruation symbolized womanhood, and its absence affected self-perception. Many felt regret over husbands’ unmet needs or viewed sex as a duty, while some preferred celibacy. Lubricants, hormone therapy, adult films, and partner communication were seen as helpful for enhancing sexual life.
Hinchliff et al (2010), UK^17^	12 women 48–60 years	Qualitative study Face to face interviews	To examine the changes in sexual activity women can experience at menopause and the impact on psychological well-being.	High	Most women reported decreased sexual desire post-menopause, influenced by marital issues, parental responsibilities, and partners’ health, though some experienced increased desire in new relationships. Many felt distress over sexual changes and concern for partners’ unmet needs.
Hinchliff et al (2018), UK^40^	Total: 1,084 Women (n = 680) men (n = 404). Age ranged 50–90 years old.	Qualitative open-ended comment boxes	To explore self-reported sexual difficulties of older women and men.	High	Most participants reported sexual difficulties; some adapted through masturbation or considered extramarital relationships. Post-menopausal women expressed concern for partners’ unmet needs, though those aged 70–79 found life fulfilling without sex. GPs were the main help source, though some found them unhelpful.
Rocha et al (2014), Brazil^41^	25 women aged 40–55 years living in Urban area. N=8 professional healthcare in family health strategy.	Qualitative approach. Semi-structured interviews.	To comprehend how women aged between 40 to 55 years live their sexuality in the climacteric period and to understand how the local health system is organized to ensure comprehensive care.	Moderate	Women noted bodily changes during the climacteric affecting sexuality. Sexual activity was valued for health and relationships, but societal taboos made discussions uncomfortable Confusion between climacteric and menopause was common, with symptoms like hot flashes and reduced desire. Some embraced aging with self-care, while others struggled with sexual identity. Partner support and health service guidance were seen as vital, while cultural and psychological factors influenced perceptions of sexuality.
Ramakuela et al (2015), South Africa^42^	women between the ages of 40 years and above	Phenomenological approach. Focus groups interview.	To explore and describe culture, sex and menopause among rural women of Vhembe District Limpopo provinence, South Africa.	High	Strong cultural beliefs discourage premenopausal and postmenopausal women from sex, associating it with health risks like bulging tummies, leading to suppressed desire. Participants reported sexual changes—vaginal dryness, painful, unenjoyable sex, and stress from feeling obligated to satisfy partners. Lack of interest in sex sometimes led men to seek extramarital relationships, increasing wives’ risk of STIs.
Miller (2019), US^43^	39 women aged 35–91 years	Qualitative Life story interviewing methods	How do women’s views and experiences with dating and sex change as they age?	Moderate	Caregiving duties and lack of partners limited dating opportunities for some women, while others felt increased sexual agency and saw new partners as enhancing their sexual lives. Menopause boosted pleasure for some by removing pregnancy concerns. Older women were generally less open to casual sex than middle-aged women.
Lodge and Umberson (2012), US^44^	17 long-term married couples. (Range: 50–86 years)	Qualitative In-depth interviews	To examine how gender and age as cultural discourses and social structural locations intersect to shape the sexual experiences of mid- to later life married couples.	Moderate	All couples experienced reduced sexual frequency due to ageing and partner health issues, but many noted improved quality, linked to comfort, freedom from parenting stress, and emotional intimacy. Some women reported increased desire post-menopause, and midlife couples were more likely to feel upset by sexual changes. Emotional connection was often prioritized over sex.
Hughes (2013), US^45^	27 women aged 50—80 years	Qualitative study Semi-structured interview	To learn more about how mid-to-late-life women approached communication about sexual health with their health care providers and what factors impacted their perceptions of their abilities to do so.	Moderate	Women are willing to discuss sexual health with providers if they display interest and nonjudgmental attitudes, though confidentiality concerns remain. The patient-provider relationship is crucial for open communication about sexual issues.
Hughes and Lewinson (2015), US^46^	28 women, 45+ years	Qualitative approach Semi-structured interview	To understand aging women’s perspectives about communicating with providers about sexual health using integrative model of behavioral prediction as a theoretical lens.	High	Most women have a positive attitude toward discussing sexual health with providers, though some, agreeing with their husbands, view it as a private matter unrelated to other health issues. A few hold a negative view on communicating about sexual health with providers.
Kirkman et al (2015), Australia^47^	Total:4 Two women and two men. born between 1946 and 1965, living in a rural area.	Qualitative research. In-depth interviews	To investigate the experience of rural baby boomers in friends-with benefits relationships.	Moderate	Two middle-aged women expressed sexual desire and a willingness to challenge societal norms by seeking new relationships, showing less interest in practicing safer sex.
Watson et al (2017), US^48^	14 women aged 64–77 years.	Qualitative study Semi-structured in-depth interview	To investigate how aging impacts women’s views of their own sexuality as well as sexual relationships and what sex means to them at this point in their lives.	High	Women felt aging did not hinder their sexuality but saw it as a time to renew desire. While intercourse was less enjoyable due to partner issues, they valued intimacy. Women in new relationships reported greater enjoyment compared to those in marriages.
Araújo et al (2013), Brazil^49^	40 climacteric women aged 45–65 years.	Qualitative descriptive study Interview method	To investigate the social representations of the sexual life of climacteric women assisted at public health services.	Moderate	Some women found sex unsatisfying due to partner misunderstandings, body changes, ageing, and family responsibilities, often engaging passively to please their partners. However, others experienced peak sexual fulfillment at this stage, aided by self-care, companionship, new partners, life stability, and freedom from pregnancy concerns. Feeling understood, loved, and respected was key to igniting desire, while some women found peace in abstaining from sex, focusing on religious activities and grandchildren.
Ribeiro Araújo et al (2017), Brazil^50^	Total: 7 6 women, 1 male between 65 and the 78 years old	Qualitative approach. semi-structured interview	To identify the needs of the elderly in view of their sexuality to subsidize the construction of an educational technology	Moderate	Participants reported less frequent but pleasurable sex compared to youth, with elderly women experiencing challenges like pain and vaginal dryness. Communication about sex was notably lacking.
Vidayanti and Retnaningsih (2020), Indonesia^51^	12 Postmenopausal women aged 50–60 year.	Phenomenological approach in-depth, face-to-face, semi-structured interviews.	Describe the sexual experience of women during the postmenopausal period which can be addressed as a proper platform of sociocultural relevance.	High	Most participants reported negative changes in sexual function post-menopause, including reduced frequency, vaginal dryness, discomfort, fatigue, and difficulty achieving orgasm. Some husbands were understanding, leading to reduced physical intimacy but sustained communication and romance. Many participants adopted a healthy lifestyle to enhance their sex life.
DeLamater et al (2019), US^52^	24 women, aged 55–80,	Qualitative study Face-to-face interviews.	To explore changes in women’s sexuality in later life, defined here as aged 55–80, and, how women experience these changes.	High	Most participants noted a decline in sexual desire post-menopause, seen as normal by some, while others experienced increased desire with new partners. Decline was linked to partners’ health issues, personal health, menopause, and long-term relationships. Some maintained sexual frequency due to its importance, while others abstained. Aging and weight gain affected self-perception, with adaptation strategies including masturbation, movies, sex toys, lubrication, and visits to sexuality centers.
Ford and Chamratrithirong (2012), Thailand^53^	44, 22 men and 22 women aged 53–57 years.	Qualitative research. In-depth interviews	To examine, through a qualitative study, views of changing sexuality among married Thai adults in their early to mid-fifties.	High	Most participants reported a decline in sexual frequency with age post-menopause, citing increased responsibilities, decreased stamina, and menopausal changes like difficulty with orgasm and maintaining erections. Concerns were raised about extramarital sex and HIV risk.
O’Mullan et al (2019), Australia^54^	8 midlife women aged 45–64 years,	Qualitative study In-depth interview	To explore the factors and mechanisms that enable Australian women aged 45 to 64 years to successfully negotiate safer sex practices in new relationships.	Moderate	All participants felt vulnerable to STIs and actively sought to reduce risk. Healthcare providers played a key role by offering nonjudgmental spaces for discussing sexual concerns. Women took responsibility for initiating safer sex conversations and carrying condoms or negotiating safety with partners.
Fileborn et al (2018), Australia^55^	Total: 53 Australian women (n = 23, 43.4%) and men (n = 30, 56.6%) aged 60 years and older.	Qualitative study Interview methods	To explore older adults’ knowledge of and use of STI prevention and safer sex.	High	Women viewed condom use as essential, especially with new partners, and some insisted on STD testing before unprotected sex. A few felt STIs were unrelated to their age. Some were embarrassed to discuss safer sex and felt judged for raising the issue. For those with vaginal dryness, condom use was reported as painful.
Feltrin and Velho (2014), Brazil^56^	99 women Aged 35–82 years old. It includes interview with 15 doctors residents studying gynaecology and undergraduate medical students.	Ethnography analysis Interviews and observations.	To understand the experience of sexuality after menopause among a group of women, through their life experiences, considering the construction of meanings attributed to this phase by doctors and patients in the gynaecologist’s office environment.	High	Some sexual issues, even those linked to menopause, trace back to early life experiences, with sexuality viewed as taboo and many difficulties attributed to lifelong hormonal changes. Dissatisfaction and loss of pleasure were often related to life events and partner health, though physicians framed these issues within menopause’s medicalization. Many saw sex as a duty to retain their husbands, influenced by negative social expectations. Menopause was positive for some, offering freedom from pregnancy and more sexual agency. Women valued husbands’ sexual interest for relationship maintenance and accepted desire discrepancies as normal. Parental attitudes also impacted menopause and sexuality.
Syme et al (2019), US^57^	Total: 373 50+ years participants (50% women, 50% men). 55% between ages 50-+70	Qualitative study. Using online survey	To address How do mid- and later life adults define their sexual well-being.	High	Some women viewed menopause as a major challenge to sexual wellness and desire. Masturbation and foreplay were emphasized more by women than men. Women in mid- to later life referenced emotions and age-related aspects of sexuality at least twice as often as men.
Stahl et al (2019), US^58^	16 women 55- +75 years	Phenomenological approach In-depth interviewing	To explore the experience of women aged 57–91 to better understand sexual pleasure over a lifetime.	Moderate	Women challenged social norms to embrace their sexuality, exploring new activities and pleasure sources, supported by social changes like leaving home, divorce, and women’s liberation. Solo sex and masturbation provided pleasure, while all valued intimacy in relationships. Some reported difficulty finding partners.
Thomas et al (2021), US^59^	36 women aged 60 and older	Qualitative Individual interviews and focus groups	To understand the ways in which moderate libido affects the lives of older women.	High	Women saw sex as important and sought to understand ‘normal’ aging effects on sexuality. Most felt distressed by low libido and guilt over unmet partner needs, fearing reduced activity would make them feel less attractive or old. A few embraced decreased libido positively, focusing on other life aspects.
Thomas et al (2019), US^22^	39 women aged 45–60 years	Qualitative Individual interviews and focus groups	To explore the role of body image in sexual function and satisfaction in a racially diverse group of women 45–60 years of age.	High	Many women saw feeling attractive as a key motivator for sexual activity, though some found it less important with age. Dissatisfaction with body weight was more common among white women, while some felt confident and attractive regardless. A supportive partner helped some feel sexually attractive, though one reported jealousy affecting her self-view.
Thomas et al (2020), US^60^	36 women aged 60 and older	Qualitative Individual interviews and focus groups	To explore factors that contribute to low libido among sexually active women 60 and older using a qualitative approach.	High	Vaginal symptoms were common, with women adapting through hormones, lubricants, or pelvic exercises. Partner erectile dysfunction reduced libido, leading women to adjust behaviors, but communication about it was often challenging. Fatigue, pain, stress, caregiving, and body image concerns also decreased libido, though some grew less concerned with appearance over time.
Thomas et al (2017), US^61^	39 women aged 45–60	Qualitative Individual interviews and focus groups	To better understand both the sexual function outcomes that were most important to them and the types of treatments they would prefer.	High	Women sought to address issues like low desire, sexual pain, vaginal dryness, and decreased orgasmic response, prioritizing emotional over physical outcomes. Feeling attractive motivated them, and many preferred behavioral treatments over medications due to side effect concerns, with some favoring group-based support.
Thomas et al (2018), US^62^	39 Women aged 45–60	Qualitative Individual interviews and focus groups	To better understand midlife women’s lived experiences of changes in sexual function with aging.	High	Women experienced frustrations with negative changes like reduced frequency, low libido, dryness, and orgasm difficulties, linked to menopause or psychosocial factors. Partner health issues also lowered libido. Adaptations included adjusting sexual behaviors, lengthening foreplay, new positions, and using aids. Many valued connection and intimacy over physical outcomes, with some finding sex more meaningful or satisfying than in youth due to improved communication and long-term relationship fulfillment.
Harder et al (2019), UK^63^	Total: 4,418 comments were eligible. Women (aged 50–75).	Qualitative study Using a questionnaire	To examine sexual activity, functioning, and satisfaction in a large sample of postmenopausal women from the UK Collaborative Trial of Ovarian Cancer Screening.	High	Most women were sexually inactive due to lack of a partner, caregiving, health, menopause, and life responsibilities. Some viewed sex as benefiting their spouse. HCPs were rarely consulted; lubricants and Hormonal Replacement Therapy (HRT) helped a few. Adaptations included showing love, masturbation, and nonpenetrative sex. New partners boosted satisfaction for some, while 9% accepted decreased sex as natural aging, with commitment growing.
Paine et al (2019), US^64^	48 midlife women and 16 men in 16 lesbian marriages and 16 straight marriages. range from age 40 to 60 years	Qualitative study in-depth interviews	To examines how married straight and lesbian women understand sexual changes in midlife.	Moderate	Most women, both lesbian and straight, reported a decline in sex due to health changes, menopause, and weight gain, with lesbians particularly affected by shared menopausal experiences and caregiving. Many viewed reduced sex as natural with age and relationship length. Straight couples saw an opportunity to improve intimacy after children left home, while some straight women felt stressed and struggled with communication about sex. Communication was key for improvement, with lesbian couples engaging more intensively. A few couples from both groups reported contentment without sex.
Hyde et al (2011), Ireland^15^	25 Postmenopausal women between 42 to 63 years.	Qualitative study. Individual semi-structured interviews and focus group	To present women’s accounts of heterosexual experiences in the context of menopause.	High	Few women cited biological reasons for sexual changes, focusing more on psychological factors and parental responsibilities for decreased desire. Some noted little or no change post-menopause, with positive experiences often linked to new partners. While desire was low, many enjoyed sex once initiated. Desire discrepancies caused tension for some, though understanding partners eased guilt over declining sexual activity.
Ussher et al (2015), Australia^16^	Study1: women aged 41–56 years old. Study2: 39 women aged 20–71 years.	Qualitative semi-structured in-depth interviews face-to-face	To present a critical examination of women’s experiences of sexuality during and after the menopausal transition.	High	Older women prioritize relationship quality over biomedical factors to maintain desire, often continuing coital sex despite pain, leading to distress. Many feel liberated from menstruation and fertility, gaining sexual confidence, though bodily changes can affect self-perception. Partner acceptance, new or long relationships, and an empty nest renew sexual activity for some, while others find long-term relationships reduce it. Partner health issues and negative attitudes toward menopausal changes decrease desire. Feeling desired encourages continued sexual activity, with alternatives like mutual masturbation, oral sex, lubricants, and hormone creams commonly used, especially among lesbians.
Faccio et al (2018), Italy^65^	Total: 146 people participated. 48 experts, 48 women diagnosed with hypoactive sexual desire (41–60) and 50 ordinary people.	Qualitative Face to face interview	To investigate how professionals, women diagnosed with hypoactive sexual desire disorder and ordinary people, both men and women, signify this phenomenon in the Italian context.	High	Decreased desire in women stems from both physiological changes and long-term relationships, which can reduce attraction. Menopausal bodily changes sometimes lead to depression, with women attributing these changes to hormones and seeking management. Communication with partners is emphasized, with gynecologists and psychologists seen as valuable sources of support.
Shakour et al (2018), Iran^66^	Total: 22 12 postmenopausal aged between 45–65 years and 10 men	Qualitative Semi-structured interview	To assess reproductive health needs in men and women as the first and basic step in educational planning.	High	Women expressed needs for reproductive health support, focusing on menopausal symptoms like anxiety and palpitations and seeking suitable treatments. Reduced sexual pressure, health issues (theirs or their partner’s), and financial problems were cited as factors in decreased libido. They suggested a dedicated center for consulting on sexual health issues.
Utian and Maamari (2014), Canada, Sweden, US, UK^67^	70 postmenopausal women Between 40–75 years	Qualitative Focus groups	The impact of postmenopausal vaginal atrophy and women’s coping strategies were evaluated through international focus groups.	Moderate	Women’s views on menopause ranged from seeing it as an achievement to an unnoticed stage. Vaginal atrophy impacted them physically, relationally, and emotionally; some used lubricants, while others felt embarrassed. Most lacked knowledge and saw it as a normal part of aging, often not seeking help, with willingness to seek support varying by personality.
Reyhani et al (2018), Iran^68^	30 women 38–59 years	Qualitative In-depth semi structured interview	To determine the women’s emotional and psycho-social perceptions caused by reproductive changes in the middle-age period.	High	Women reported that fertility changes affected their feminine identity and shape, with decreased sexual attraction diminishing their sense of femininity. Some questioned their role post-fertility, while others accepted the transition as a normal and maturing phase.
Lewis et al (2020), UK^69^	Total: 19 9 men 10 women aged 40–59	Qualitative Face to face interview	To identify factors shaping STI risk perceptions and practices among midlife individuals either contemplating or having sex with new partners following the end of a long-term relationship	High	With menopause-related fertility loss, some women felt less need for condoms or safer sex, though they would test if concerned about a partner’s fidelity. Embarrassment and partner resistance hindered condom use. Women prioritized children’s emotional needs after divorce, often above their own, and feared ageist judgment when seeking safer sex guidance.
Morison and Cook (2015), New Zealand^70^	8 women aged 40–69 years	Qualitative Face to face semi-structured interview	To contribute to knowledge about barriers to safer sex behaviours for midlife women living in New Zealand.	High	Participants noted that healthcare professionals rarely initiated discussions on safer sex, and all admitted to unprotected sex in new or casual relationships, sometimes influenced by alcohol. Condoms were seen as reducing pleasure and romance, with traditional gender roles hindering safer sex negotiations.
Smith (2015), US^71^	10 women aged 45–56 years.	Qualitative In-depth interview	To understand middle-age urban African American women’s experiences with HIV-related sexual risk behaviors and to identify the sexual protective strategies they employed to reduce their risk for HIV infection.	High	About half of the participants had positive attitudes towards sexual protection and condom use, with some citing religious beliefs as motivation. However, some lacked STI knowledge or engaged in sexual risk behaviors. Sociocultural factors, such as the incarceration of Black men and resistance to condom use, contributed to sexual risks among older African American women. Most women felt empowered to make positive sexual decisions and negotiate condom use, but some experienced gendered power dynamics and domestic violence, which hindered their ability to negotiate safer sex.
Shifren et al (2019), US^72^	75 overall women aged 45 years or older and postmenopausal. 36 women diagnosed with GSM	Qualitative Interviews and focus group	To describe menopausal women’s experience of GSM in their own words to better understand the syndrome and its impact on women’s lives.	High	The study aimed to develop a tool based on women’s experiences with Genitourinary Syndrome of Menopause. Women reported symptoms like vaginal dryness, irritation, pain during sex, and burning afterward, which negatively impacted their quality of life, relationships with partners, and sense of femininity.
Chen et al (2023), China^73^	21 married postmenopausal women aged between 45–70 years. Who experience natural menopause.	Phenomenological qualitative methods Face-to-face semi-structured interview	This study aimed to explore perceptions, experiences, and coping strategies related to sex among postmenopausal women in China.	High	Most respondents believed postmenopausal sex was necessary, though 28.6% held negative views based on traditional Chinese cultural beliefs. Women reported symptoms like decreased sex drive, vaginal dryness, pain, and orgasm difficulties, influenced by life stressors and partner health. Many felt guilt or worry, while some enjoyed the freedom from menstruation and pregnancy concerns. Concerns about partner infidelity and vaginal dryness affecting relationships were common. Sex was often avoided due to shame, hindering discussions or help-seeking. Some viewed sex as an obligation, while others found exercise and communication helpful in improving their sexual lives.
Hayfield et al (2024), UK^74^	Total=71 British women who self-defined as perimenopausal, menopausal, or postmenopausal Ages ranged from 37	Qualitative online open-ended qualitative survey	Focused on women’s experience of their interpersonal relationships during peri/menopause	High	Some partners were perceived as lacking understanding, while others were supportive. Women often viewed their declining sexual interest as a personal issue that affected their relationships, leading to feelings of guilt. Some men blamed women for these changes and sought extramarital relationships. True support came from partners who understood how peri/menopause affected their intimacy, allowing them to face challenges together
Rafiei et al (2024), Iran^75^	17 postmenopausal women aged between 50–62, and N=7 healthcare experts	Qualitative study Semi-structured interviews	Aimed to elucidate the concept of enriching the sexual life of women post-menopause.	High	Maintaining sexual relationships during menopause strengthens romantic bonds and benefits the couple and family. Key factors include sexual awareness, mutual participation, and attention to each other’s needs to improve intimacy. Effective strategies for enhancing post-menopausal sexual life involve preparation, identifying stimuli, maintaining focus, and using sexual fantasies. Positive actions, like focusing on appearance, create a better atmosphere for intimacy, while expressing sexual desires fosters deeper understanding and satisfaction between spouses.
de Boer (2023), Netherland^76^	11 Dutch menopausal women aged between 46–61	Interpretative phenomenological empirical analysis In-depth interviews	Explores the meaning of sexual identity work in menopause.	High	Women reported that menopause-related physical and emotional changes, like vaginal dryness, sensitive skin, and hot flashes, decreased sexual desire, making encounters less frequent or unpleasant. Some believed sex should be for self-pleasure, not just to satisfy others, as non-pleasurable sex compromised their sense of self. Many sought to reconnect with their bodies and explore new sexual preferences, particularly regarding orgasms. Women reflected on how physical changes, such as weight gain and sagging breasts, influenced their sense of attractiveness, yet prioritized self-acceptance over external validation. Some experimented sexually with others, distancing themselves from traditional sexual norms.
Ayalon et al (2021), Israel^77^	23 women and 24 men aged 60 and older	Qualitative approach Interview	To examine the reflections of older men and women on sexual functioning in later life.	High	Many women initially focused on sexual changes in their partners rather than themselves, with some not noticing any changes or even reporting improvements. Partner health issues were identified as challenges to sexual desire and contact. Most women reported a decline in sexual desire and expressed concern about how this might negatively affect their partner’s well-being.
Wellings et al (2023), UK^27^	23 women aged 45–59	Qualitative study part of the mixed method Semi-structured interviews	To explore the associations between sexual satisfaction, activity, and function among midlife women, using both quantitative data from a national survey and qualitative interviews to understand women’s perceptions of influences on their sexual activity.	High	Most women did not view age as a major factor in sexual activity, except when health issues affected sexual response. Few linked menopause to a decline in sexual quality, and many appreciated the freedom from menstruation and pregnancy risk. Some reported depression due to decreased sexual activity, while long-term relationships led to dulled sexual desire. Women in new relationships often had improved sexual experiences. Concerns about aging and losing attractiveness were common. A healthy relationship enhanced sexual experiences, while sexual inactivity sometimes led to separation. Discussing sexual difficulties was difficult, and fatigue, caregiving, and work demands often reduced energy for sex.

### Data Analysis

Fifty-three studies were included in this literature review. Identifying the studies’ data, including their aims, designs, locations, and sampling, quality assessment results, as well as their main findings, was organized into tables, see Table 1. The analysis used the data extraction form in this table. The synthesis process followed the seven iterative stages of meta-ethnography as outlined by Noblit and Hare.^78^ After identifying and selecting relevant studies, we conducted an in-depth reading of each article to understand their aims and key findings. Each article was reviewed for content and meaning to inform the development of a preliminary coding framework. Key concepts and themes were extracted using first-order data (participants’ quotations) and second-order interpretations (authors’ analysis and themes). The first reviewer independently coded each study, while a second reviewer checked the coding for completeness, and a third reviewer reviewed the coding for accuracy and consistency.

After that, we examined how the studies related to one another by identifying recurring concepts and translating themes across the data set. This involved reciprocal translation, where similar concepts were grouped together and refined. An index article was selected to guide the initial coding framework, which was then applied to the rest of the included studies. Second-order interpretations (authors’ themes and interpretations) and illustrative first-order data were organized to support cross-study comparison.

Through iterative discussion, we translated key findings into each other to generate third-order interpretations. This involved comparing themes to determine whether they were reciprocal (conceptually aligned), refutational (contradictory), or if a line of argument synthesis was needed. The synthesis primarily followed reciprocal translation, highlighting shared patterns across studies. The final phase involved developing a coherent and interpretive synthesis that captured the overarching themes while preserving the contextual richness of women’s experiences of sexuality during midlife and menopause.

## Results

### Study Characteristics and Main Findings

Studies were conducted in a wide range of countries including the USA, UK, Australia, Brazil, Ireland, Italy, Taiwan, Turkey, South Africa, New Zealand, Iran, Lebanon, Nigeria, Indonesia, Netherlands, Israel, and China. One study was conducted in four countries including Canada, Sweden, the US, and the UK. Twelve studies included both women and men, three studies involved women and healthcare providers, and one study included women, healthcare providers, and men. The remaining studies focused solely on women. The age range of the participants was between 35 and 91 years. The sample size of the included studies ranged from 4 to 4418. Most studies collected data through semi-structured in-depth interviews, followed by focus groups, observations, or qualitative surveys with written comments. The interview was conducted mainly face-to-face, while some studies conducted it through telephone, Zoom or Skype.

Different methods were adopted for the analysis, including thematic, content, framework, and template analysis. Some used Tesch’s and open coding method, grounded theory, interpretive phenomenological, ethnographic materials, deductive and inductive, material and discursive framework analysis. The characteristics of the 53 included studies, including study place and design, participant age, and key findings, are summarized in Table 1.

The findings of the review are presented in three main themes: Physical symptoms and cultural influences on sexuality during menopause and midlife, emotional, psychological, and relational responses, and adapting to sexual changes during the midlife and menopause transition. These themes will be discussed in the following sections.

### Theme 1: Physical Symptoms and Cultural Influences on Sexuality During Menopause and Midlife

A key focus of this theme is on the physical symptoms of menopause related to sexual health and well-being, partner health issues, and the impact of cultural views and societal perceptions on menopause and sexual health.

### Menopausal Symptoms and Sexual Health

Many factors can impact women’s experience of sexual changes throughout midlife and menopause, including biological ones. It was shown in the findings of this review that women experience vaginal dryness and pain during sexual activity after midlife and menopause.^15^,^42^,^51^,^61^,^62^,^72^,^73^ Vaginal atrophy was reported also as a symptom that affects women in the postmenopausal stage.^67^ In some studies, women have trouble reaching orgasm after menopause.^51^,^61^,^62^,^73^ Some studies noted that vaginal dryness prevents some women from using safer sex measures such as condoms, which makes them vulnerable to sexually transmitted infections.^55^

Several studies from this review highlighted the decline in sexual desire and frequency of sexual activities in women after menopause and midlife.^9^,^17^,^34^,^39^,^44^,^50–53^,^57^,^61–64^,^73^,^77^ Although many of them reported a decrease in sexual desire or frequency of sex during menopause, one study noted that only a few participants experienced such changes in their sexual desire^15^ and another study found that most participants did not report changes in sexual drive related to menopause.^27^ Similarly, the results of a study involving 23 women over the age of 60 showed that four women did not experience any changes in their sexual activities with age.^77^ This suggests that sexual symptoms are experienced or perceived differently by different groups of women throughout midlife and menopause transition.

It is important to note that some women attribute their sexual changes to the aging process rather than to menopause itself.^44^,^49^,^53^ Therefore, they may believe their symptoms are a natural part of aging rather than directly connected to menopause. This leads to a blurring of the distinction between menopause and aging, with some women viewing this change as inevitable rather than a result of hormonal shifts during the menopause period. However, one study found that no women believe aging negatively impacts their sexuality.^48^ This also indicates the variation in how women recognize the cause of their symptoms, which can have implications on their ability to seek help. Additionally, some women attribute the changes in their sexual lives to their own health issues.^52^,^55^,^63^,^66^

It is common for women to report changes in their sexuality during or after menopause, such as decreased libido or physical discomfort; however, according to this review these changes did not diminish their enjoyment of sexual activity entirely. Numerous studies have shown that intimacy is fulfilling and even more pleasurable despite changes in sexual activities.^50^,^62^,^64^,^77^ The findings demonstrate that the biological changes of menopause do not uniformly diminish sexual fulfillment; rather, women’s interpretations of these changes vary based on health status and personal attitudes toward ageing. Recognizing diversity in women’s experiences allows for a more individualized and culturally sensitive approach to care.

### Impact of Partner’s Health

Women often described how their sexual interest was influenced by the sexual difficulties experienced by their male partners. Several studies in this review suggest that women’s decreased drive may be influenced by their partner’s health or sexual issues.^9^,^17^,^37^,^44^,^48^,^52^,^56^,^62^,^63^,^66^,^73^ Erectile dysfunction in male partners is one of the most common sexual problems that affect women’s sexual desire and activities in midlife.^62^,^64^ Indeed, a recent study found that older women primarily expressed their distress over their partners’ declining desire and performance when reflecting on the impact of sexual changes on their well-being.^77^ This highlighted the importance of examining the male partner’s health when studying women’s sexual function during and after menopause. This relational perspective moves the discourse beyond individual pathology to a more systemic understanding of midlife sexuality.

### Cultural Perceptions, Medicalization, and Knowledge Gaps in Sexual Health

Some studies reported that healthcare providers often focus primarily on physiological symptoms and medical treatments, with less attention given to the relational and psychological aspects of sexual health. This emphasizes the medicalization of menopause, which frames it predominantly as a biological condition requiring medical intervention. The lack of consideration for broader psychosocial factors leaves some women feeling unheard when discussing their sexual health concerns. One study noted how doctors tend to emphasize biological phenomena over sociocultural context when discussing women’s sexual complaints after menopause.^56^ While most of these women in their studies attributed their sexual dissatisfaction to trauma and stressful life events, their physicians only discussed it from a biological perspective. In a similar vein, another study found that women believed that their menopausal sexual changes were caused by hormonal changes and needed to be treated, emphasizing the Western medicalization of menopause and its influence on how women perceive this life stage.^65^

The cultural and social views of menopause and sexuality seem to affect the way menopausal women feel about their sexuality. Two studies conducted in African countries reported that menopause is considered a marker of sexual retirement for women.^26^,^42^ In these studies, many participants held onto cultural traditions, which inhibited their ability to understand menopause accurately. Several participants shared traditional views, including a fear that sexual activity after menopause might cause folk pregnancy or abdominal rupture, which could lead to death, reinforcing a reluctance to engage in sexual activity after menopause. A Chinese study found that their culture expects postmenopausal couples to sleep separately and elders with sexual desires are regarded as lustful and morally inappropriate.^73^ This emphasized the asexuality view reported in Iranian and Lebanese cultures about menopausal women,^35^,^36^ where the latter were considered hopeless at this stage for engaging in or enjoying sexual activity. While some cultures place expectations on women to maintain sexual activity in later life,^32^ others view the cessation of sexual activity as normative or appropriate.^26^ These contrasting views highlight the diversity of cultural norms shaping women’s experiences.

The lack of knowledge about menopause also appears to negatively influence women’s perception of it. One study noted that Iranian women’s perceptions of menopause were heavily influenced by medicalization, seeing it as a serious illness and receiving information from friends instead of reliable sources.^33^ Similarly, Chinese women attribute their knowledge of menopause and sexual activity to their elders.^73^ Consequently, women struggle to find accurate information and avoid seeking medical advice, which exposes them to a wide range of misconceptions about menopause.^41^ Indeed, some studies demonstrate a lack of knowledge about sexual health practices during midlife and menopause and report that some women engage in risky sexual behaviors and some of them consider the lack of fertility due to menopause as a reason for not using safe sex measures.^69–71^ Additionally, some studies have shown that some men resist using condoms, coercing their partners into unprotected sex despite the risks of sexually transmitted infections,^69^,^71^ while traditional gender role perceptions, such as prioritising men’s preferences and avoiding conflict, were reported as significant barriers to negotiating safer sex.^70^ These intersecting influences underscore the need to move beyond a biomedical or individualistic view and instead adopt a culturally sensitive approach that recognizes how power, knowledge, and cultural expectations shape women’s sexual agency in midlife.

### Theme 2: Emotional, Psychological, and Relational Responses

The theme demonstrates the emotional, psychological, and relational changes women can experience during menopause, particularly changes in self-perception, body image, and reframing their sexual rights.

### Body Image and Loss of Femininity

According to the findings of this review, women viewed looking attractive as an important factor in motivating their sexual desire during menopause.^9^,^22^,^61^ Throughout menopause and midlife, some women notice changes in their bodies as they age, such as gaining weight, wrinkles, and changing breast shapes. These physical changes worry many women in their menopausal transition about their beauty and attractiveness leading to experiencing low sexual desire,^16^,^17^,^22^,^27^,^33^,^52^,^60^,^64^,^68^,^76^ despite their partners’ assurances about their attractiveness.^16^,^22^

Beauty standards imposed by culture may have contributed to women’s sensitivity to concerns about attractiveness as evidenced across cultures. This emphasizes the importance of societal and cultural perceptions about gender expectations and norms for women while going through menopause. While women acknowledged the presence of societal beauty standards, some described learning to navigate or resist these expectations as they aged. For example, one study noted that black women in the US reported greater confidence than white women, with many of them attributing their confidence to their partners’ acceptance and support.^22^ Similarly, another study reported that some women stressed the importance of self-acceptance and self-attractiveness during the menopause transition.^76^ Therefore, it is crucial to examine how women feel about their bodily changes during the menopause transition to understand the impact these changes have on their sexuality.

### Psychological Pressures, Emotional Strain, and Relational Dynamics in Sexual Desire

Women in their menopausal transition experience psychological pressures that affect their sexual desires. One of the main reasons for declining sexual desires and activities during midlife was caring for children and older adults.^9^,^15^,^17^,^26^,^27^,^43^,^49^,^53^,^63^,^64^ Additionally, life stressor events, other responsibilities, and financial issues were reported to be reason for declining sexual desire in their menopausal transition.^56^,^66^,^73^ In a recent study conducted among midlife women, it was found that the husband’s instability in his job was reflected in the woman’s lack of sexual desire due to her feelings of insecurity.^27^ There is evidence, however, that a decrease in responsibilities and an increase in financial stability during midlife can lead to enhanced sexual desire.^16^,^44^,^49^,^64^ This illustrates how psychological pressures can significantly diminish sexual desire since women in midlife and menopause tend to prioritize others’ needs over their own.

The lack of sexual interest in women seemed to have a negative impact on their psychological well-being. Many women reported emotions like depression, frustration, being upset and distress as a result of the decline in their sexual activities.^17^,^27^,^44^,^77^ There were other women who expressed worry about their partner’s unmet needs.^17^,^34^,^40^,^59^,^73^,^74^ Some menopausal women questioned their role in their husbands’ lives and highlighted the emotional impact of sexual difficulties during menopause on their sense of self-worth and relationship dynamics.^39^ These negative feelings expressed by women in midlife and menopause can stem from societal or relational expectations that women continue to engage in sexual activities, even when their own desire has diminished. Additionally, it stressed the importance of sexual activity during menopause and midlife for their relationship as evidenced in many studies.^41^,^52^,^56^,^59^,^62^,^75^

According to the above studies, some women felt guilty about not meeting their partners’ sexual needs, which led to them engaging in sexual activities just to satisfy their partners,^17^,^39^,^41^ representing sexual activity as a marital obligation. Indeed, some women view the fulfilment of a spouse’s sexual needs as a religious duty in marriage, particularly in Islam.^32^,^34^,^36^ This creates another pressure on the woman to engage in sexual activity while experiencing low desire during midlife and menopause. A study found that women’s submissions to their husband’s sexual needs were to maintain their families’ stability for the sake of their children,^36^ demonstrating the importance of women’s submission to conservative societies. Such dynamics can negatively impact the quality of life and intimate relationships;^65^,^72–74^,^77^ unresolved sexual dissatisfaction or unmet expectations may lead some partners to seek fulfilment outside the relationship, resulting in infidelity or, in some cases, divorce,^27^,^74^ indicating the importance of sexual intimacy to the health and longevity of a relationship.

The dynamic of relationships among couples appears to play a significant role in determining sexual desire and continuity of activities. The duration of a relationship has been demonstrated to influence women’s sexual desires. Long relationships were linked to declining sexual desire among midlife and menopausal women.^16^,^27^,^52^,^65^ However, some midlife women report that being in long relationships positively affects their sexual desires.^16^,^62^ Additionally, relationship quality was determined to be a significant determinant of sexual desires among midlife and menopausal women. The experience of relationship problems and difficulties reduces the desire for sexual activity among midlife and menopausal women.^17^,^38^,^63^ Nevertheless, midlife women who experienced a good relationship reported improvements in their sexual desires.^35^,^49^ Moreover, the partner’s ability to understand and support their wives during the menopausal transition enhances and improves their sexual desire.^16^,^22^,^62^ On the other hand, the partner’s negative attitude toward menopause and bodily changes and their lack of understanding of their wives, adversely affect a woman’s sexual desire.^16^ This shows how the dynamics of a relationship can have a significant impact on women’s sexual desires.

### Positive Reframing and Renewed Sexual Identity

Menopause can be considered a positive stage for some women. The decline in fertility during menopause and a sense of free-from menstruation and contraceptives allow women to engage in sexual activities without worrying about pregnancy.^16^,^27^,^35^,^36^,^43^,^49^,^56^,^73^ This sense of freedom is reported to be an important factor for menopausal women to be more satisfied and experience more enjoyment in their sexual activities. This demonstrates that physiological changes in sexual function after menopause do not necessarily negatively affect women’s lives.

Menopause and midlife are reported to be the best times to renew sexual desire and activities for some women. Having a new partner and exploring new sexual relationships at this time reported renewing their sexual desires.^15–17^,^27^,^43^,^47–49^,^52^,^63^ This shows the ability of midlife and menopausal women to have more sexual agency during this period. Some studies described how older women challenge societal norms to express themselves as sexual beings.^58^ This type of sexual agency allows women to challenge societal taboos that stigmatize women’s sexuality, especially in menopause and midlife. Although there were more reports of women seeking new sexual relationships in Western societies rather than other cultures, no studies were conducted in Muslim countries to report this, where women cannot pursue extramarital relationships without divorce or remarriage.

While some studies demonstrated how midlife and menopausal women challenge societal norms to express their sexual desires, some studies reported that menopausal women can re-negotiate their sexual activities. They expressed how menopausal women challenge the patriarchal gender and discuss their sexual dissatisfaction or even reject it for the first time.^32^,^33^ In a similar vein, one study shows how women asserted that sex should be for self-pleasure and not to satisfy others, distancing themselves from traditional social norms after menopause.^76^ Additionally, the empowerment of women in their midlife has led to positive decisions about their sexual health and confidence in discussing safer sex with their partners,^71^ reflecting on their positive sexual health.

### Theme 3: Adapting to Sexual Changes During the Midlife and Menopause Transition

There were different approaches by which women try to adapt to the sexual changes during midlife and menopause. Several studies show that women tend to use a lubricant, local hormone cream, or Hormonal Replacement Therapy (HRT) to cope with vaginal dryness.^16^,^39^,^52^,^60^,^62^,^63^,^67^ Others mentioned some behaviors such as masturbation, adopting a healthy lifestyle, watching movies that portrayed sexual activity, pelvic floor exercises, lengthening foreplay, sex toys, trying different sexual positions, and creating positive and motivating atmosphere to enhance sexual life.^17^,^34^,^39^,^51^,^52^,^58^,^60^,^62^,^63^,^75^ However, Iranian and Lebanese studies emphasized the importance of beauty and appearance by having cosmetic surgery to restructure their genital and facial anatomy and look younger,^32^,^35^,^36^ highlighting how cultural expectations influence adaptation strategies for menopausal women.

Communication with the partner can play a crucial role in how midlife and menopausal women adapt to sexual changes.^73^ Studies have shown that women who communicate openly about their sexual concerns with their partners are better able to adjust to their sexual lives after midlife and menopause.^34^,^51^,^62^ A study noted that lesbian women reported more intense communication with their partners than straight women, showing the variation in communication with other sexual identities.^64^ However, some women reported having difficulty initiating sexual communication with their partners, whether for cultural reasons or because such conversations were met with negative reactions, making communication unsuccessful or unhelpful for them,^34^,^50^,^60^ indicating the differences in the cultural norms regarding sexual issues discussion. In a similar vein, it has been reported that some midlife and menopausal women feel embarrassed to discuss aspects of sexual health with their partners, such as safe sex practices,^55^,^69^ which could lead to a higher risk of sexually transmitted infections.

Seeking help from healthcare professionals is reported as a way to deal with sexual difficulties during midlife and menopause. Some women reported seeking help from a gynecologist,^36^,^65^ healthcare providers,^17^,^63^ or visiting the sexuality health center,^52^ while some women lacked a sexual consultation center and reported their need for this service.^66^ However, midlife and menopausal women seek help and communicate their sexual health concerns differently. Some women showed openness to seeking sexual help if their providers showed interest and provided a non-judgmental environment.^45^,^46^ Nevertheless, some women neglect sexual issues in their lives either because they are ashamed or do not view them as a problem.^46^,^67^,^73^ Additionally, some studies noted that midlife and menopausal women fear being judged negatively if they seek healthcare providers’ assistance with safe sexual practices,^55^,^69^ therefore, avoiding seeking help for their sexual health. This emphasized the importance of healthcare providers in facilitating the discussion of sexual health and well-being for women in their midlife.

## Discussion

The findings from this review highlight a complex interplay between the physical symptoms of menopause, partner health issues, cultural views, and relational context that together shape women’s experiences of sexual health and well-being. Women during and after menopause face unique challenges when it comes to maintaining their sexual well-being, as biological changes and external factors interact to affect their intimacy and satisfaction. This discussion focuses on how these biopsychosocial aspects affect women’s sexual health while going through menopause.

The findings revealed that many women in menopause and midlife experienced some changes in sexual function, including a decreased sex drive, vaginal dryness and pain, and difficulties achieving orgasm. Similar symptoms have been reported in previous studies,^79^,^80^ manifesting the biological impact of hormonal changes in sexual function during and after menopause. The findings of this review also highlight the crucial role that partner health plays in shaping women’s sexual experiences during menopause and midlife. This is in line with studies that found that women engaged in significantly less sexual activity and experienced decreased sexual desire, arousal, and orgasm when their partners developed erectile dysfunction.^81^,^82^ Like the current review, these studies emphasize that sexual intimacy is strained not only because of the woman’s physical symptoms but also because her partner is unable to engage in sexual activity, emphasizing the need to take into account the partner’s sexual health when assessing a woman’s sexual health in midlife and menopause.

The findings of this review indicate that cultural views play a significant role in shaping women’s experiences of sexuality during menopause. In many non-Western cultures such as Nigeria, Lebanon, and China, menopause is often associated more with the end of a woman’s sexual identity, where sexual activity is closely linked to reproduction.^26^,^36^,^73^ However, there have also been reports of asexual views among older women in Western cultures such as the UK,^83^ reflecting a complex cultural context. This stigma could arise from cultural norms that associate sexual attractiveness with youth, marginalizing older women who are no longer considered sexually desirable in society. Increasing medicalization of ageing may contribute to the negative perception of sexuality in older women in Western cultures, where older women are seen as primarily patients managing health decline, rather than as individuals with sexual agency.^16^ Furthermore, patriarchal attitudes continue to emphasize male desires, ignoring or disregarding older women’s sexual needs. Therefore, it is important to promote positive views about menopause and ageing and to encourage healthcare providers to treat sexual health concerns sensitively so that women experiencing menopause or midlife feel empowered to express their needs.

The findings show that body image issues play a significant role in many women’s declines of sexual interest during menopause and midlife. During menopause, women often experience physical changes such as weight gain, loss of muscle tone, and wrinkles that alter their sense of attractiveness and femininity. This is in agreement with both quantitative and qualitative studies that have shown that body image concerns negatively impact women’s sexual interest during the menopause transition.^84–86^ These studies highlight women’s emotional and psychological struggles while feeling a decrease in femininity as a result of physical bodily changes, resulting in a lack of sexual desire. Additionally, supportive partners may improve sexual desire, while criticism or lack of understanding about bodily changes can increase emotional distance, further diminishing women’s sexual desire.^22^ This highlights the need for cultural shifts regarding ageing and body image, as well as the need for open communication within relationships.

This review shows that increased daily life stressors and responsibility for children and family members diminish women’s sexual desires and activities during menopause and midlife. These results confirm the findings of the Seattle Midlife Women’s Health Study, which found that stress and life obligations significantly impacted sexual desire in women during the transition to menopause.^14^ This highlights the importance of considering biopsychosocial aspects for healthcare providers when assessing midlife and menopausal women’s sexual desires and activities, including both biological changes, psychological stress, and social pressures women face. Moreover, the findings of this review revealed that many menopausal and midlife women feel regret and guilt when they are unable to fulfil their partner’s sexual needs and feel obligated to fake their sexual desire, which underscores gender differences in expectations around sexuality. These results were found in both Western and non-Western societies,^82^,^87^ reflecting how patriarchal norms influence women’s perceptions of responsibility for their partner’s desires in diverse cultural contexts. Therefore, women during the menopause transition should receive supportive counselling that helps them focus on their own sexual health and well-being.

The findings of this review unveiled positive experiences reported by menopausal women and midlife to their sexual satisfaction. The attitude of women toward menopause depends on their expectations of menopause’s advantages and disadvantages, which is in line with previous studies.^88^ Women in this review found menopausal time to be a source of sexual agency. The increased confidence and acceptance of self, along with challenging social expectations and gendered norms, has allowed women to enjoy intimate relationships into their midlife and beyond.^16^,^52^ In this regard, encouraging women to view menopause as a time for exploring, enjoying, and establishing their sexual agency as well as self-empowerment during this phase of life is vital.

The findings of this review illustrate women’s resilience and the diverse strategies they use to cope with physical and emotional changes during menopause. The use of HRT was one of the methods women considered to deal with their sexual difficulties. This approach was discussed more in Western cultures, thus emphasizing the need to raise women’s awareness of it in both Western and non-Western cultures. The provision of education about HRT will empower women to make informed treatment decisions. The review also highlights attitudes toward seeking help for sexual issues in midlife and menopause. However, many women report that cultural or personal barriers can restrict their confidence when seeking help with sexual issues. Therefore, healthcare professionals should actively create a warm and safe environment for women to discuss sexual concerns during menopause, offering encouragement, education, and support regardless of whether they actively seek assistance.

## Strengths and Limitations

This meta-ethnography provides insight into women’s intimacy and sexual life experiences in their midlife and menopausal transition. Rather than focusing solely on postmenopause, it covers the entire midlife and menopause continuum and provides a comprehensive understanding of midlife experiences. In this review, women’s biopsychosocial experiences are explored concerning their transition to menopause and midlife sexual changes, which is valuable for healthcare providers to know. Additionally, the review’s cross-cultural inclusivity contributes to its relevance across a wide range of contexts.

Limitations of this review include its focus on English-language sources, which means that research in other languages has been excluded, and valuable insights might have been missed. Among the studies focusing on menopause transition, only five explicitly mentioned the participants’ menopausal stages. Results that omit the menopausal stage may leave gaps in understanding specific menopausal experiences. Therefore, this review cannot be certain about the exact menopausal stage. However, careful consideration has been given to ensure the representation of the findings by keeping the “menopausal transition” term. Additionally, nearly half of the studies recruited their participants from healthcare centers, which may introduce selection bias. Women attending clinics might have more severe symptoms or greater health awareness compared to those who do not seek clinical care, potentially limiting the generalizability of the findings to all women experiencing menopause. Other studies, however, involved community settings and public spaces. By limiting recruitment to health clinic visitors, the experience of other women who did not attend clinics may be ignored. The exclusion of studies involving specific subgroups, such as women experiencing surgical or premature menopause and marginalized populations, may have limited the inclusivity and generalizability of our findings. These groups may encounter distinct sociocultural, medical, or psychological challenges that were not captured in this review. Future research should aim to explore these experiences to broaden the understanding of sexual health across the menopause continuum. Another limitation of this review is that the majority of included studies originate from Western countries. Despite an inclusive search strategy, there is a notable lack of research from non-Western and developing countries, limiting the findings’ cultural diversity. Additionally, the exclusion of grey literature may have overlooked valuable insights. Future research should include it to enrich the biopsychosocial understanding of women’s sexuality during menopause, especially across diverse cultural contexts.

## Conclusion

The finding of this review shows that women’s experiences of sexual change in menopause and midlife are diverse and complex across cultures. It demonstrates how sexual experiences in midlife are influenced by multiple factors, primarily the interaction of biological factors such as hormonal changes, psychological factors such as self-esteem, and social factors including cultural expectations and partner relationships. The review highlights the importance of healthcare providers considering biopsychosocial factors when addressing sexual issues during midlife and menopause. This approach enables the development of individually tailored treatment plans that address hormonal changes, psychological challenges, and relational concerns. It also empowers women to make sense of, adapt to, and embrace the sexual changes they experience during midlife and menopause, fostering greater confidence in viewing sexuality as a natural part of this life stage and beyond. Future research should prioritise women’s sexual experiences in non-Western and developing countries to inform care approaches that are culturally relevant and globally applicable.

## Data Availability

The data supporting the findings of this review are derived from the articles cited in the manuscript, which are publicly available through their respective journals or repositories. Any additional data are available from the corresponding author upon reasonable request.

## Relevance to Clinical Practice

This meta-ethnography provides valuable insights into women’s sexual experiences throughout the midlife and menopause continuum. In examining these experiences through a biopsychosocial lens, it emphasizes the complex interplay between biological, psychological, and social factors. This review is highly relevant to healthcare providers since it emphasizes the need to provide care in a more holistic, culturally sensitive manner. Increasing women’s awareness and understanding about menopause, sexual health, and overall well-being can help them navigate midlife transitions more confidently through education, support groups, and reliable healthcare resources. Healthcare providers such as general practitioners, gynecologists, and nurses can also assist with sexual health concerns, and facilitate open, non-judgmental discussions. Providing women with positive, personalized care that is specifically tailored to their needs can help make discussions about menopause more relatable and promote healthy living. A shift in practice can improve the quality of care, enhance provider-patient relationships, and support women’s overall health in midlife and menopause.
